# Targeting Breast Cancer Stem Cells

**DOI:** 10.7150/ijbs.76187

**Published:** 2023-01-01

**Authors:** Lu Zhang, Wenmin Chen, Suling Liu, Ceshi Chen

**Affiliations:** 1 Fudan University Shanghai Cancer Center & Institutes of Biomedical Sciences; State Key Laboratory of Genetic Engineering; Cancer Institutes; Key Laboratory of Breast Cancer in Shanghai; The Shanghai paracrine Key Laboratory of Medical Epigenetics; Shanghai Key Laboratory of Radiation Oncology; The International Co-laboratory of Medical Epigenetics and Metabolism, Ministry of Science and Technology; Shanghai Medical College; Fudan University, Shanghai 200032, China.; 2 Key Laboratory of Animal Models and Human Disease Mechanisms of Chinese Academy of Sciences and Yunnan Province, Kunming Institute of Zoology, Kunming 650201, China.; 3 Kunming College of Life Sciences, the University of the Chinese Academy of Sciences, Kunming 650201, China.; 4 Jiangsu Key Lab of Cancer Biomarkers, Prevention and Treatment, Collaborative Innovation Center for Cancer Medicine, Nanjing Medical University, Nanjing 211166, China.; 5 Academy of Biomedical Engineering, Kunming Medical University, Kunming 650500, China.; 6 The Third Affiliated Hospital, Kunming Medical University, Kunming 650118, China.

**Keywords:** Breast cancer stem cell, Epithelial-Mesenchymal Transition, Tumor microenvironment, Therapeutic strategies

## Abstract

The potential roles of breast cancer stem cells (BCSCs) in tumor initiation and recurrence have been recognized for many decades. Due to their strong capacity for self-renewal and differentiation, BCSCs are the major reasons for poor clinical outcomes and low therapeutic response. Several hypotheses on the origin of cancer stem cells have been proposed, including critical gene mutations in stem cells, dedifferentiation of somatic cells, and cell plasticity remodeling by epithelial-mesenchymal transition (EMT) and the tumor microenvironment. Moreover, the tumor microenvironment, including cellular components and cytokines, modulates the self-renewal and therapeutic resistance of BCSCs. Small molecules, antibodies, and chimeric antigen receptor (CAR)-T cells targeting BCSCs have been developed, and their applications in combination with conventional therapies are undergoing clinical trials. In this review, we focus on the features of BCSCs, emphasize the major factors and tumor environment that regulate the stemness of BCSCs, and discuss potential BCSC-targeting therapies.

## Introduction

The mammary epithelium is composed of two main cellular lineages: luminal epithelial cells and myoepithelial cells. Accumulated evidence from both mice and humans showed that these two epithelial cells are derived from common ancestors, namely the mammary epithelial stem cells 1, 2. The mammary stem cells (MaSCs) were observed to reside in the outer basal compartment and are responsible for the production of basal myoepithelial progeny cells (unipotent stem cells) or basal inner luminal progeny cells (multi/bi-potent stem cell) 3, 4. The MaSCs are heterogeneous and comprise multiple diverse subsets to meet the developmental needs including protein C receptor (PROCR)-expressing cells, transcription factor B-cell lymphoma/leukemia 11b (Bcl11b)-expressing cells, etc. 5-7.

Interestingly, a correlation between breast cancer subtypes and distinct stem cell populations is observed. Based on the expressions of estrogen receptor (ER), progesterone receptor (PR), and human epidermal growth factor 2 (HER2/ERBB2), breast cancer is simply classified into three major subtypes: luminal type with hormone receptor positive/ERBB2 negative (70% of patients), ERBB2 positive (15-20%), and triple-negative (TNBC, tumors lacking all three standard molecular markers; 15%) 8, 9. Generally, the luminal subtype is ER-positive and the TNBC subtype largely overlaps with the basal-like subtype. It has been found that luminal cancers have similar profiles to mature ER^+^PR^+^ luminal cells 10. In addition, ER^-^PR^-^ luminal progenitors share similar expression patterns with basal-like breast cancers 11. Molyneux et al. found breast cancer 1 (BRCA1) deletion in mouse mammary epithelial luminal progenitors grows tumors that resemble human BRCA1 breast cancers, supporting a derivation of the majority of human BRCA1-associated tumors from luminal progenitors 12.

Surgery is usually the first choice for breast cancer treatment. Most women may receive additional treatment, including chemotherapy, hormone therapy, radiation or targeted therapy. More than 90% breast cancer don't occur metastasis at the time of diagnosis 13. Nevertheless, it is estimated that nearly 30% of women with early-stage breast cancer will develop metastatic disease 14, and the median survival for these women ranges from 8 to 36 months 15.

Conventional therapies for breast cancer are insufficient to eliminate all cancer cells in the tumors, especially cancer stem cells (CSCs), which can lead to recurrence and drug resistance 16. BCSCs are characterized by their ability to initiate tumors from tiny numbers and are insensitive to chemo- or radio-therapies 17, 18. Analogous to stem cells, BCSCs exhibit a delicate equilibrium between self-renewal and differentiation to maintain tumor homeostasis 19. On the one hand, they differentiate into various and heterogeneous cancer cells, forming the tumor bulk 20, 21. On the other hand, they perpetuate the stem cell pool by self-renewal throughout cancer progression 22, 23. In response to environmental stimuli, when non-CSCs are eliminated by traditional chemo- or radio-therapies, BCSCs survive oxidative stress or DNA damage due to their cell dormancy and enhanced ability for DNA damage repair and drug efflux 24. During tumor metastasis, BCSCs first infiltrate the surrounding mesenchyme or enter the circulation by EMT. Then, BCSCs undergo a reciprocal program called mesenchymal-epithelial transition (MET) to form sizable metastatic colonies at distal organ sites 25. Considering that BCSCs are closely related to cancer prognosis and progression, numerous efforts have been made to characterize and eradicate BCSCs.

In this review, we comprehensively introduced the origin of BCSCs and summarized the latest research progresses on BCSCs, including features, functions, and targeting strategies. Finally, we discuss future research directions in this field.

## The origin and heterogeneity of BCSCs

The observation that BCSCs share many traits with MaSCs indicated that these two cell populations might have a common origin. For example, CD44 and PROCR label both MaSCs and BCSCs 5, 26-28. Several signaling networks essential for stemness maintenance are common in both MaSCs and BCSCs, including the NOTCH, Hedgehog, and Wnt pathways.

CD133 is initially discovered in hematopoietic stem cells and bone marrow-derived circulating endothelial progenitors 29-31. Then, CD133^+^ cancer cells were reported to be featured by strong self-renewal potential and contributed to vasculogenic mimicry (VM) in TNBC 32. In BRCA1-associated breast cancer, CD133^+^ BCSCs are distinct from CD24^-^CD44^+^ BCSCs, suggesting BCSC populations are heterogenous 33.

Integrin α6, also known as CD49f, may be the only biomarker present in more than 30 different populations of stem cells including embryonic stem cells, embryonic neuronal stem cells, hematopoietic stem cells, and cancer stem cells 34. Therefore, a hypothesis that mutagenesis in normal stem cells results in CSC formation has been proposed 35. For instance, Van Keymeulen et al. discovered that mutation of phosphatidylinositol-4,5-bisphosphate 3-kinase catalytic subunit alpha (PIK3CA) in mammary luminal stem cells leads to luminal or basal-like breast tumors. In contrast, BRCA1-basal-like breast cancers might originate from basal stem cells 36. Liu et al. found that BRCA1 participated in the differentiation of stem cells. When BRCA1 is lost, uncontrollable accumulation of stem cells occurs, and finally, a small number of stem cells missing BRCA1 develop into cancer 37.

Another speculation on the origin of cancer stem cells is EMT, an essential program for embryonic development. The first work on the relationship between EMT and stemness was conducted in the mammary gland. microRNA (miRNA)-200 family comprises five members (miRNA-200a, -200b, 200c, -141 and -429) and all members were markedly downregulated in cells with EMT 38. EMT induced mitochondrial fusion during asymmetric division of MaSCs via miR-200c-peroxisome proliferator-activated receptor-γ coactivator 1 alpha (PGC1α)-mitofusin (MFN1) pathway. The fused mitochondrial was asymmetrically separated to stem cell-like progeny with enhanced reactive oxygen species (ROS) scavenging and glutathione synthesis capacities. Downregulation of the EMT/miR-200 axis promotes the expansion of mammary stem cells by transforming growth factor beta (TGF-β)-induced asymmetric division 39. Evidence has shown that during tumor progression, whether in the very beginning stage or the final metastatic phase, cancer cells activate EMT transcription factors (EMT-TFs) to favor their proliferation and survival. For example, Wellner et al. showed that zinc finger E-box binding homeobox 1 (ZEB1), a vital activator of cancer metastasis, promoted tumor-initiation capacity by repressing stemness-inhibiting miRNAs 40. Poornima et al. overexpressed EMT-TF SLUG in CD24^+^/CD44^-^ MCF-10A cells and MCF-7 breast cancer cells and found a small population of CD24^-^/CD44^+^ stem-like cells emerged 41. Their work proved that the association of EMT and stem cells was adopted by normal epithelial cells and the corresponding derived neoplastic cells. However, whether cells gain pluripotency by EMT remains controversial. Ocana et al. discovered that the EMT activator paired related homeobox 1 (PRRX1) cooperates with EMT-TF Twist1 in all EMT-related characteristics, but it suppressed stemness in breast cancer 25, 42.

Acquisition of a partial EMT phenotype or a hybrid E/M state seems more critical for stemness and tumorigenicity of breast cancer cells. Liu's study found the first evidence of an association between BCSC heterogeneity and EMT. They discovered that a CD24^-^/CD44^+^/ALDH^+^ BCSC population with intermediate EMT characteristics had the most vital capacity for stemness and tumorigenesis 43. Another study on integrin subunit beta 4 (ITGB4)-positive BCSCs showed that this BCSC subpopulation was in an intermediate E/M state and induced a worse five-year probability of relapse-free survival 44. Indeed, more and more evidence showed that the hybrid E/M state led to strong stemness and poor prognosis and was independent of cellular origin 45-48. Cornelia et al. isolated highly malignant CD104^+^/CD44^hi^ breast cancer cells expressing both epithelial and mesenchymal markers. Complete transition to a mesenchymal state decreases their capacity for stemness and tumorigenesis 49. Satiwik et al. employed a mechanism-based mathematical modeling framework to demonstrate that intermediate E/M phenotype enrichment is coupled with enhanced stemness and stemness is more likely to develop in intermediate E/M phenotypes than in “pure” epithelial/mesenchymal phenotypes 50. Meredith et al. analyzed three single-cell clones with intermediate E/M state and demonstrated core-binding factor subunit beta (CBFβ) was responsible for stabilizing and maintaining metastatic ability. Their results showed that EMT score alone was not associated with survival while CBFβ showed predictive value for survival outcomes 51. Recently, nuclear factor erythroid 2-related factor 2 (NRF2) functioned as a stability factor for intermediate E/M cells. NRF2-EMT-NOTCH network signaling is spatially coordinated near the leading edge during collective cancer migration 52.

Another model currently predominates in explaining the origin of CSC is the stochastic model because CSC and non-CSC interconvert. Thus, CSC cannot be isolated by cell-sorting methods based on intrinsic features 53. It is believed that cancer cells commonly sustain equilibria in the proportion of cells, and every cell owns an equal probability of initiating tumor growth 43, 54. For instance, Wang et al. showed the existence of distinctive CSC populations and bidirectional inter-conversion between non-CSC and CSCs occurred stochastically 55. Besides breast cancer, stochastic model can also apply to other solid and non-solid tumors, including lung adenocarcinomas and lymphoblastic leukemias 56-58. Like the hierarchical model, the stochastic model is composed of retro-differentiating cancer cells into stem-like cancer cells 59. However, the stochastic model primarily addresses genetic heterogeneity without consideration of potential phenotypic variations within the genetically homogenous tumor cell population.

Genetic and epigenetic alterations and abnormal activation of signaling pathways can promote the malignant transformation of normal stem cells. Human stem cells with the surface marker phenotype Lin^-^CD10^-^CD24^-^PROCR^+^CD44^+^ were identified in normal mammary epithelium and breast carcinomas. Notably, both PROCR and CD44 are target genes of the Wnt pathway 5, 60. The altered Hippo signaling pathway also confers self-renewal and metastatic ability to BCSCs. The signaling effectors transcriptional co-activator with PDZ-binding motif (TAZ) and yes-associated protein (YAP) have been shown to bind to the promoters of mammary stem cell signature genes to induce BCSCs 61, 62, and high expression of TAZ was detected in CD44^+^CD24^-^ BCSCs 63. YAP/TAZ amplification correlates with the poor prognostic outcome and increased therapeutic resistance 64. In addition, gene fusions were found in TAZ, NF2, and LATS1/2 in lung cancer 65, and TAZ-CAMTA fusion or YAP-TFE3 fusion is proved to be the initiating mutation in a vascular cancer 66. These gene fusions induce the Hippo pathway hyperactive and drive tumor initiation and proliferation* in vivo* 67.

In addition, several studies have reported that some mammary epithelial cells in a dormant state might give rise to BCSCs under specific circumstances. For example, Guo et al. found that co-expression of SRY-box transcription factor 9 (SOX9) and SLUG in differentiated luminal cells was sufficient to induce stem cell-like properties, such as EMT activation and metastasis-seeding ability 68. Similarly, Dravis et al. found that the binding of SOX10 to genes related to EMT or to genes that regulate neural crest cell identity promoted the stem-like features of mammary tumor cells 69. Recent studies employing single-cell sequencing with chromatin accessibility indicated that, due to epigenetic regulation, some epithelial cells expressed both luminal and basal signature genes within the mammary epithelium, due to epigenetic regulation, which might explain the multipotent capacity of basal cells observed upon transplantation 70.

CSCs in tumors exhibit very heterogeneous metabolic states, and each CSC has an adaptable metabolism. Somatic stem cells 71, embryonic stem cells 72, 73, and induced pluripotent stem cells 74 are all reported to increase their glycolysis activity to maintain their stem cell features. Angela et al. compared the CSCs to the parental and benign precursor cells and found that CSCs with increasingly glycolytic phenotypes are more adaptable to specific microenvironmental conditions 75. Luo et al. illustrated how metabolic or oxidative stress modulated the BCSC dynamic state. The transition between mesenchymal-like state and epithelial-like state relied on redox metabolism change. For epithelial-like BCSCs, they exhibit strong antioxidant capacity due to NRF2 hyperactivity. Oxidative stress transited epithelial-like BCSCs to mesenchymal-like state 76. Patricia et al. also observed similar phenomenon in pancreatic cancer, supporting that some CSC populations are dependent on oxidative metabolism 77.

## BCSCs are involved in multiple biological behaviors of breast cancer

**Figure 1:**
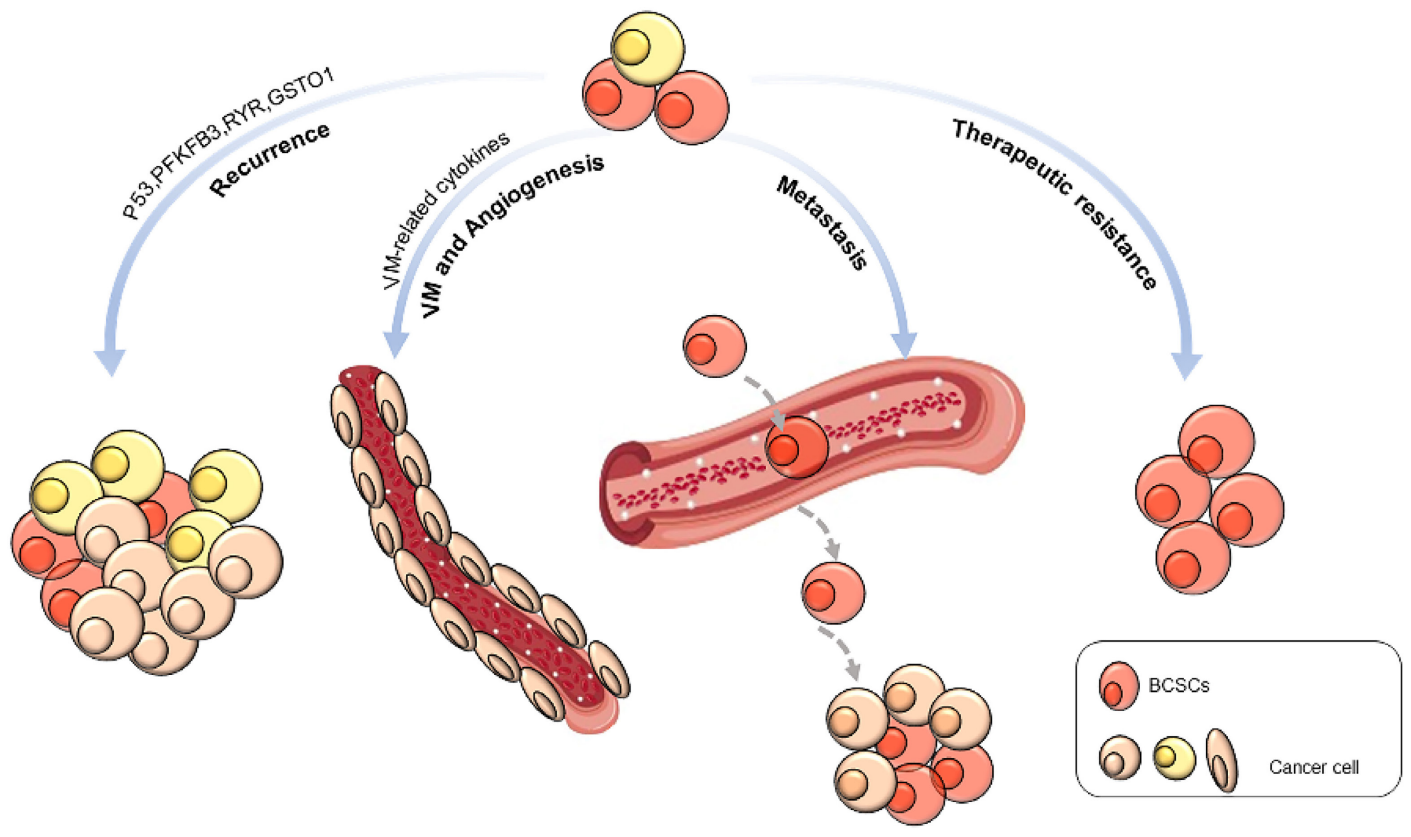
Biological behaviors of BCSCs.

BCSCs are engaged in the physical behaviors of cancer, including recurrence, metastasis, vasculogenic mimicry, angiogenesis, and therapeutic resistance (**Figure 1**).

### Tumor recurrence

Emerging evidence suggests that BCSCs promote tumor recurrence, leading to poor prognosis 78. The silencing of p53 promotes the division of BCSCs, increasing their renewal and contributing to tumor recurrence 79. Inhibition of ryanodine receptor (RyR1) and glutathione S-transferase omega 1 (GSTO1) expression cleared off chemotherapy derivational BCSC enrichment and postponed cancer recurrence 80. Abnormal expression of 6-phosphofructo-2-kinase/fructose-2,6-biphosphatase 3 (PFKFB3), a gene associated with tumor recurrence, can be suppressed by autophagy activation, keeping BCSCs in a resting state 81.

### Vasculogenic mimicry and angiogenesis

Recent studies indicated that BCSCs might contribute to tumor-associated angiogenesis by VM or trans-differentiation 82. For example, Bussolati et al. isolated CD24^-^/CD44^+^ BCSCs and cultured them to differentiate into an endothelial lineage with endothelial markers and properties in the presence of vascular endothelial growth factor (VEGF). They also observed intratumor vessels of human origin in transplanted tumors 83. Using a 3D reconstructed image, Sun et al. provided direct evidence of the relationship between BCSCs and VM formation. The CD133^+^ BCSCs were observed to line VM channels, and breast cancer cells encircled VM channels. They also observed a close correlation between BCSC proportion and VM in invasive breast cancer 84. In addition to differentiation into endothelial cells, BCSCs might provide VM-related cytokines, such as Nodal protein, to support angiogenesis 85. Tumor endothelial marker 8 (TEM8) is highly correlated with VM, and TEM8 coincidentally induces VM and promotes stemness through the Ras homolog family member C (RhoC)/rho associated coiled-coil containing protein kinase 1 (ROCK1)/smad family member 5 (SMAD5) axis 86.

### Tumor metastasis

CSCs are closely related to tumor metastasis 87. BCSCs are more metastatic than other types of breast cancer cells, which was realized by increasing the expression of cell metastasis and migration-related proteins while significantly reducing the level of adhesion proteins 88. In BCSCs, a subset of the cell population expressing CD44v and epithelial splicing regulatory protein 1 (ESRP1) has a stronger lung metastasis ability 89. Six homeobox 2 (Six2) promotes the expression of SRY-box transcription factor 2 (SOX2) and NANOG, which induces stem cell characteristics and increases TNBC metastasis 90. The inflammatory cytokine interleukin-8 (IL8) promotes BCSC metastasis by activating C-X-C motif chemokine receptor (CXCR1/2) 91. Transforming growth factor beta 1 (TGF-β1) significantly increases the number of BCSCs in MDA-MB-231 cells and promotes breast cancer metastasis to the liver 92. C-C motif chemokine ligand 2 (CCL2) enhances CSC self-renewal and expansion through the signal transducer and activator of transcription 3 (STAT3) and neurogenic locus notch homolog protein 1 (NOTCH-1) signaling pathways and promotes breast tumor growth and metastasis 93, 94. Stromal cell-derived factor-1 (SDF-1/CXCL12) activates the nuclear factor-κB (NF-κB) pathway to increase the proportion of BCSCs and promote the metastasis of MCF-7 cells 95. Consistently, Shan et al. showed that CXCL12 overexpression induced MCF-7 cells to form a BCSC phenotype through the Wnt/β-catenin pathway, thereby enhancing metastasis 96.

Inactivation of tumor suppressor genes and activation of oncogenes also regulate the metastatic potential of breast cancer stem cells. Knockout of p21 in a breast tumor model inhibited the self-renewal and lung metastasis of BCSCs 97. Silencing AKT serine/threonine kinase 2 (AKT2) reduces the invasion and colony formation abilities of BCSCs, which are mediated by the Twist/mTOR signaling axis 98. Inhibition of RhoC dramatically reduces the probability of lung metastasis of SUM149 99. Extracellular signal-regulated kinase 2 (ERK2) promotes BCSC self-renewal and lung metastasis, and knockout of ERK2 significantly inhibits colony formation and mammosphere formation 100. Deletion of sirtuin 1 (SIRT1) accelerates the degradation of PRRX1 and sequentially activates the transcription of krüppel-like factor 4 (KLF4) and aldehyde dehydrogenase 1 (ALDH1), thereby inducing BCSCs and lung metastasis 101. GD3 synthase (ST8SIA1) is highly expressed in BCSCs, and inhibiting this synthase reduces tumor growth and metastasis by eliminating BCSCs 102. OTU deubiquitinase 7B (OTUD7B) deubiquitinates lysine-specific demethylase 1 (LSD1) to decrease histone H3 lysine 4 (H3K4)/H3K9 methylation, thereby sustaining breast cancer metastasis potential 103.

Overexpression of miR-7 in BCSCs decreases endothelial cell adhesion molecule (ESAM) expression by targeting RelA and inhibits tumorigenesis and distant lung metastasis 104. miRNA-628 directly targets SOS Ras/Rac guanine nucleotide exchange factor 1 (SOS1) to inhibit the migration and invasion of BCSCs 105. Specifically, hypoxia-mediated upregulation of miRNA-210 induces BCSC migration by blocking E-cadherin expression 106.

LncRNA MALAT-1 is upregulated in BCSCs, and it promotes self-renewal, migration, and invasion of BCSCs by regulating SOX2 107. Linc-ROR promotes the proliferation and invasion of BCSCs by inhibiting the abnormal overexpression of critical factors such as SMAD family member 2 (SMAD2) and alpha-smooth muscle actin (α-SMA) in the TGF-β signaling pathway 108. LncRNA NR2F1-NAS1 binds to the 5'-UTR of nuclear receptor subfamily 2 group F member 1 (NR2F1) and recruits polypyrimidine tract binding protein 1 (PTBP1) to enhance the translation of NR2F1. The latter inhibits the transcription of ΔNp63, thereby inhibiting the MET of BCSCs and ultimately inducing the metastatic dormancy of cancer cells 109.

### Therapeutic resistance

Current studies have shown that BCSCs are one of the main reasons for radiation resistance and chemotherapy resistance. Yang et al. reported that aurora kinase A (AURKA) promotes forkhead box M1 (FOXM1) transcription, self-renewal, and drug resistance in BCSCs 110. The deubiquitinase ubiquitin specific peptidase 28 (USP28) can stabilize LSD1 to promote stemness and drug resistance in breast cancer cells 111. Metformin induces miR-708 expression in BCSCs and increases the chemotherapy sensitivity of BCSCs 112. Signal-induced proliferation-related protein 1 (SIPA1) promotes the expression of stem cell-related transcription factors, such as octamer-binding transcription factor-4 (OCT4), NANOG, SOX2, and B lymphoma Mo-MLV insertion region one homolog (BMI-1), by increasing the expression of SMAD2/3, resulting in chemotherapy resistance 113.

The drug resistance of BCSCs is mainly caused by the expression of transporters that cause drug efflux and the expression of high-level ALDH1 as a detoxification enzyme to metabolize anticancer substances. In addition, BCSCs have a strong DNA damage repair ability and show radiation resistance through high expression of related stemness genes and activation of antiapoptotic and antioxidant signaling pathways 114, 115.

ALDH is closely related to BCSC therapeutic resistance. One of the reasons is that it can inactivate the metabolism of chemotherapy drugs such as cyclophosphamide 78. ALDH can produce nicotinamide to achieve antioxidant function 116. Overexpression of NANOG enhances ALDH activity by activating the NOTCH-1 and AKT pathways, thereby inducing radiation resistance 117. The drug resistance of BCSCs is also driven by a high mitochondrial quality caused by ALDH activity 116.

In addition, Hippo signaling is emphasized to play a key role in mediating therapeutic resistance. For example, the hyperactivation of YAP and TAZ is indicative of resistance to paclitaxel, lapatinib, doxorubicin, or CDK4/6 inhibitors 118-120. Cysteine-rich protein 61 (Cyr61) and connective tissue growth factor (CTGF) are the transcriptional targets of TAZ/ transcriptional enhanced associate domain (TEAD). Evidence showed that they also confer paclitaxel resistance to breast cells and inhibit Cyr61 and CTGF by shRNA re-sensitized breast cancer cells to paclitaxel. Macrophage stimulating (MST) as an important component of Hippo signaling is responsible for YAP and TAZ phosphorylation and degradation. Low expression of MST protein is associated with poor prognosis in breast cancer. Pauliina et al. revealed that phosphorylation of MST1 by fibroblast growth factor receptor 4 (FGFR4) helped cell evade from mitochondrial apoptosis and become resistant to HER2/epidermal growth factor receptor (EGFR), AKT, or mTOR inhibitors 121.

The resistance of BCSCs to radiotherapy may be caused by apoptosis reduction and DNA damage checkpoint activation. Double-strand breaks in DNA damage are mainly repaired by homologous recombination (HR) or nonhomologous end-joining (NHEJ) 78, 122. Jiao et al. showed that C-C motif chemokine receptor 5 (CCR5) enhanced DNA repair in breast cancer after chemotherapy 123. The activities of checkpoint kinase 1 (CHK1) and CHK2 are also improved in BCSCs to avoid mitotic catastrophe and to repair damaged DNA 124. By activating the phosphatidylinositol 3-kinase (PI3K) and NRF2 signaling pathways, BCSCs are more resistant to drug- or radiation-induced apoptosis 125. Activated PI3K/AKT downregulates forkhead box O3 (FOXO3a) expression levels and enhances breast cancer stemness and therapeutic resistance 126. The transcription factor NRF2 has a high expression level in BCSCs to maintain a relatively low level of ROS 127. Sequestosome 1 (SQSTM1/p62) activates NRF2 expression to overcome the drug resistance of BCSCs 128, 129. Up-regulation of the expression level of nuclear-Dbf2-related 1 (NRD1), a component of the Hippo pathway will increase the proportion of BCSCs, thereby resisting the lethal effect of drugs 130.

## The tumor microenvironment (TME) regulates BCSCs

**Figure 2:**
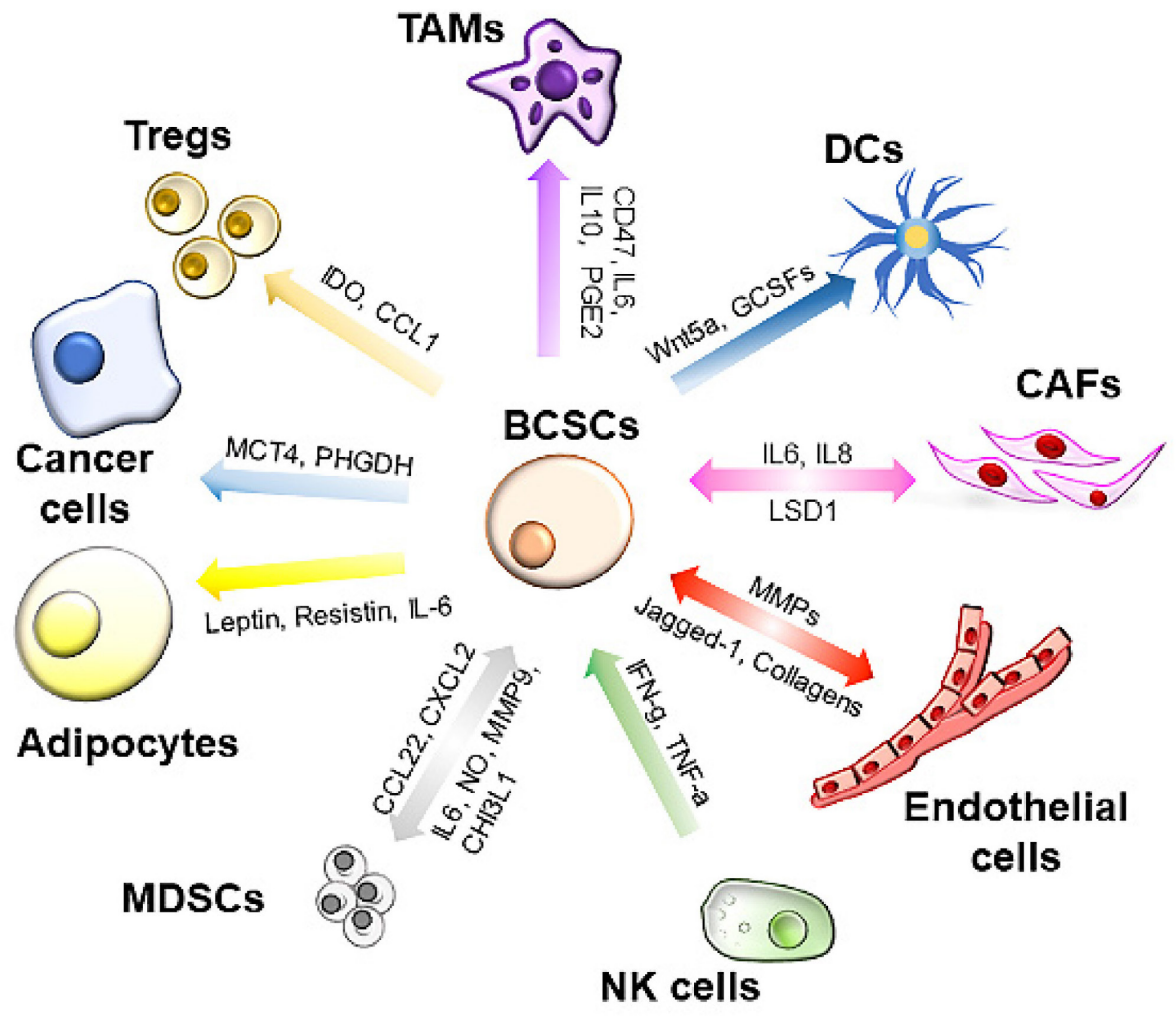
** Interplays between tumor microenvironment (TME) and BCSCs.** The TME is a complex network consisting of cellular and noncellular components. BCSCs interact with the TME through cytokines in a paracrine manner or through direct interactions. The cellular components in the TME secrete various cytokines/chemokines to support the self-renewal of BCSCs and help BCSCs escape immune attack. DCs: dendritic cells; TAMs: tumor-associated macrophages; MDSCs: myeloid-derived suppressor cells; CAFs: cancer-associated fibroblasts; MMPs: matrix metalloproteinases; GCSFs: granulocyte colony-stimulating factor; SCF: stem cell factor; PHGDH: phosphoglycerate dehydrogenase; MCT4: monocarboxylate transporter-4.

The TME consists of noncellular components, such as extracellular matrix (ECM) nutrients, metabolites and cytokines, and cellular components, including fibroblasts, adipocytes, immune cells and endothelial cells. All these components exhibit dynamic changes during cancer progression and are associated with cancer stemness (**Figure 2**).

### Hypoxia

Hypoxia is a well-known niche for CSCs. Several proteases, including serine proteases, thrombin, and matrix metalloproteinases, are activated by the acidic microenvironment around hypoxic cells and promote cancer metastasis 131-133. For example, cells with high expressions of platelet-derived growth factor (PDGF)-D and hypoxia inducible factor 1 subunit alpha (HIF-1α) exhibited more aggressive phenotypes by increasing matriptase activation 134. Thrombin was reported to be closely related to VM formation and spontaneous metastases in tumors 135. Jewer et al. discovered various transcript isoforms of NANOG, SNAIL, and Nodal. When cancer cells are under hypoxic conditions, they prefer to translate these variants to facilitate protein expression and acquire a stem cell phenotype 136. Consistently, remarkably elevated SOX2, OCT4, KLF4, and NANOG expression levels were observed after hypoxia 137, 138. The sphere formation rate under hypoxia was much higher than normal culture conditions 138. The enrichment of BCSCs was also observed in hypoxic tumors *in vivo* 139. Hypoxia-inducible factors (HIFs) play a master role in regulating malignant phenotypes. The impact of HIFs on BCSCs is mainly due to HIF-dependent hyperactivation of pluripotent factors or EMT-TFs 137. For example, Zhang et al. reported that m6A-demethylation of NANOG mRNA by HIFs, including HIF-1α and HIF-2α, significantly increased NANOG mRNA and protein expression, which further enhanced the BCSC phenotype under hypoxia 140. HIF-1α also causes abnormal nuclear translocation of FOXO3 and transcriptional activation of NANOG, which increases chemotherapy-enriched BCSCs 141. Hypoxia induces a metabolic energy change in stem cells and thus enhances stemness. It is proposed that stem cells have a unique metabolism to protect themselves from oxidant exposure. For example, upregulation of monocarboxylate transporter 4 (MCT-4) induced by hypoxia facilitates a stem-like feature of cancer cells by changing the acidic pH with increased lactic acid efflux 142. Zhu et al. identified a hypoxia-induced lncRNA KB-1980E6.3 closely related to poor prognosis, and upregulated expression of lncRNA promoted the stemness of breast cancer cells 143.

### Cytokines

There are two types of cytokines: pro-inflammatory and anti-inflammatory. Pro-inflammatory cytokines, such as IL1, IL6 and IL8 are involved in forming an inflammatory milieu and shaping pre-metastatic niches. While anti-inflammatory cytokines, including IL4, IL6, IL10, and IL13, etc., are released to prevent sustained or excessive inflammatory reactions 144, 145. Both pro-inflammatory cytokines and anti-inflammatory cytokines are closely related to BCSCs, and they show synergistic effects in drug resistance 146. In breast cancer, the induction of IL1α secretion triggers a proinflammatory environment to maintain CSCs 147. As previously mentioned, IL8 and IL6 maintain the characteristics of BCSCs by regulating CXCR1/2 and STAT3, respectively 91, 148. IL6 secreted by TAMs activates the JAK/STAT3 pathway to induce CSC enrichment and promote tumor growth 149. Blocking the NF-κB/IL8 pathway attenuates BCSC activity 150. Similarly, IL10 plays an important role in maintaining BCSCs, and the proliferation and self-renewal of BCSCs are blocked by inhibiting IL10 151. Shi and colleagues reported that the proportion of BCSCs is reduced by partial inhibition of KLF5/fibroblast growth factor-binding protein 1 (FGF-BP1) 152. CCL20 not only facilitates the expansion of BCSCs but also enhances drug resistance in TNBC 153. C-C Motif Chemokine Ligand 3 (CCL3) from cancer cells and macrophages enhances the phagocytic ability of docetaxel-induced M1 macrophages to BCSCs 154. Blocking the C-X-C motif chemokine ligand-1 (CXCL1) produced by BCSCs impedes BCSC proliferation and mammosphere formation 155. Type I interferon (IFN-I) is related to cancer cell stemness, and the expression of ALDH1A1 is increased when IFN-I signal transduction is destroyed 156.

### Cells in the tumor microenvironment

#### Carcinoma-associated fibroblasts (CAFs)

The interaction between fibroblasts and cancer cells was first observed by pathologists who noticed that the expansion of fibroblasts in the tumor context increased the levels of collagen and the abnormal expression of α-smooth muscle actin (αSMA), a phenomenon called desmoplasia 157. All fibroblasts within the TME, including normal fibroblasts and actively proliferating fibroblasts, are CAFs. Because there are functionally heterogeneous CAF groups, studies suggest a bimodal influence of CAFs on tumor progression, with both cancer-advancing and cancer-constraining effects 158, 159. Notably, tumor fibrosis occurs at the beginning of carcinogenesis, although most cancer cells arise from the epithelium. Such fibrosis in the early stage induces a chronic proinflammatory environment and directly impacts epithelial cell transformation.

Proinflammatory cytokines produced by CAFs, such as interleukin (IL) 1β and leukemia inhibitory factor (LIF), promote the inflammatory reaction in TME 160, 161. By secreting proinflammatory cytokines and proinflammatory paracrine factors, CAFs actively cross-talk with CSCs, foster the dedifferentiation of cancer cells, and support the self-renewal of CSCs 162. CAFs are also the major source for immune suppression in the TME based on the observations that CAFs hamper the recruitment of T cells to the TME and secrete immunosuppressive chemokines to promote M2 macrophages 163-165.

Activation of the IL6/STAT3/ NF-κB signaling pathway in HER2-positive PTEN-depleted breast cancer cells induces an increase in the CSC population. Inhibition of IL6- or IL8-secreting CAFs slows the growth of BCSCs 166. Recently, exosomes secreted by CAFs were reported to promote cancer cell transformation to a metastatic and tumor-initiating phenotype 167, 168. miRNAs, such as miR-21-5p, miR-143-3p and miR-378e, contained in exosomes of CAFs facilitated the EMT phenotype and dedifferentiation of breast cancer cells with increased expression of SOX2 and NANOG 169. In addition to secretory proteins and exosomes, CAFs also regulate the stemness of cancer cells via cell-cell interactions. For example, CD44 expressed on CAFs can promote the secretion of SDF-1, further motivating the stemness of CSCs 170. CD10^+^GPR77^+^ CAFs have been identified as a protumorigenic subpopulation that maintains the stem niche of CSCs 171.

#### Adipocytes

As the primary component in breast tissue, an increasing amount of evidence has shown that adipocytes enhance tumor malignancy by releasing inflammatory factors, metabolites, and exosomes. Indeed, the Body Mass Index (BMI) of women with breast cancer is higher than that of the general population at the time of diagnosis, and patients might benefit from modest weight loss after diagnosis 172. Several studies have shown that adipocytes in the microenvironment of invasive breast cancer are distinguished by their phenotypes, including smaller size and fibroblast-like shape, and molecular markers, such as collagen VI overexpression and low adiponectin expression. Thus, adipocytes that are adjacent to or communicate with cancer cells are defined as cancer-associated adipocytes (CAAs) 173, 174. CAAs have been proven to secrete more chemokines, including CCL2, CCL5 and IL6, to enhance the metastasis, stemness, angiogenesis, and proliferation of breast cancer cells 175. For example, CCL2 secreted by CAAs increased the recruitment and activation of macrophages in breast tissues to accelerate oncogenesis and angiogenesis 176. Adipocyte-secreted IL6 promoted the self-renewal of BCSCs and stimulated the invasion of cancer cells in an adipocyte/breast cancer cell coculture system 174, 177.

Multiple hormones, such as leptin, resistin and adiponectin, which are abnormally secreted by CAAs, are also responsible for the enhanced stemness of breast cancer. Bowers et al. reduced the expression of the leptin receptor (LEPR) in breast cancer cell lines and observed dramatically decreased expression of stem cell markers 178. Thiagarajan et al. further proved that leptin not only promoted BCSC survival by phosphorylating STAT3 but also transformed non-BCSCs into stem-like cells by binding to its receptor LEPR and inducing the expression of NANOG, SOX2 and OCT4 in TNBC 179. In addition, CAAs were reported to promote metastasis of TNBC via leptin signaling* in vitro* and in a PDX model 180. In the obese state, TAZ-dependent resistin expression was able to promote breast tumorigenesis 181. Furthermore, the high estrone (E1): estradiol (E2) ratio was proposed to facilitate tumor stemness properties in obese patients by activating NF-κB signaling 182. In contrast, circulating adiponectin, as a starvation hormone, was indicated to induce apoptosis and inhibit tumor cell proliferation, invasion and migration 183, 184.

The mechanisms by which obesity-induced dysfunctional adipocytes contribute to breast cancer stemness have not been clearly elucidated. Gao et al. reported that the transcription factor TAZ in adipocytes played an important role in upregulating cytokine secretion. TAZ knockdown or deficiency also impaired the tumor-supporting function of CAAs 181. Recently, Liu et al. demonstrated that cellular adaptation instead of expansion of preexisting clones is the primary driver responsible for obesity-related tumor formation. They discovered that palmitic acid, a metabolite of CAAs, enhanced the tumor initiation of breast cancer cells in a transcription factor CCAAT/enhancer-binding protein beta (C/EBPB)-dependent manner 185. In addition to cytokines and metabolites, CAAs have been reported to promote BCSCs by metabolic reprogramming. Dai et al. proved that elevated CAA-derived fatty acids fueled the stemness of breast cancer via the fatty acid oxidation (FAO)-AMP-activated protein kinase (AMPK)-YAP signaling axis. As a ROS sensor, YAP is induced by conditioned CAAs in cancer cells and sustains mitochondrial redox homeostasis 186.

#### Endothelial cells

CSCs prefer to be located near the vasculature due to the conveniences of migration and nutrition acquisition. The recent discovery that BCSCs tend to gather with the arteriolar niche in ER-positive breast cancer demonstrated a bidirectional interaction driven by lysophosphatidic acid (LPA)/protein kinase D (PKD-1) signaling between BCSCs and endothelial cells 22. The crosstalk between tumor cells and endothelial cells is essential for tumor angiogenesis. For example, cancer cells communicate with endothelial cells via cell adhesion. By gap junctions or adhesion receptors, they exchange ions and small metabolites to meet the demands for tumor proliferation 187. By analyzing single-cell RNA sequencing and protein expression profiles of primary tumor cells and lung metastases of TNBC, Rokana et al. found that the expression level of intercellular adhesion molecule 1 (ICAM1) was increased 200-fold in lung metastases. Further examination revealed that tumor cells employed ICAM1 to connect with endothelial or tumor cells to form cell clusters. ICAM1 promotes tumor cell stemness and transendothelial migration 188. Myc target protein 1 (Myct1), which is almost explicitly expressed in endothelial cells, interacts with the tight junction protein zona occludens 1 (ZO1) of cancer cells to promote a unique tumor niche for cancer angiogenesis and anti-immunity 189.

Meanwhile, the endothelial cells of the vasculature secrete soluble factors to support the growth and self-renewal of CSCs and promote drug resistance. Soluble factors such as VEGFs tend to bind with tyrosine kinase receptors and thus induce tumor cell proliferation or migration 190, 191. During progression, cancer cells in hypoxic regions recruit endothelial cells to build new vessels for oxygen and nutrition. These endothelial cells employ collagens to reconstruct the extracellular matrix and the interconnection of cancer cells. Cancer cells and stromal cells also secrete VEGF into the microenvironment to drive angiogenesis 192. Liu et al. observed that CD133 expression was closely related to VM in different breast cancer subtypes, especially TNBC. Further study confirmed that CD133-positive MDA-MB-231 cells could form tubular structures and express VE-cadherin, MMP-2, and MMP-9.

In addition, cancer cells might mimic the embryonic vasculogenesis process during metastasis. The vessel-like structures are favorable for periodic acid-Schiff (PAS) staining and negative for CD31. Notably, the VM showed perfusion capacity, and erythrocytes were found inside the VM, suggesting that they might support tumor cells in hypoxia by transporting nutrients and oxygen. In breast cancer, ALDH1 expression was associated with the formation of VM, especially in TNBC 193. For example, ALDH1A3^+^ HCC1937 cells with inducible p53 transfection showed a high capacity to form tubular structures when cultured in Matrigel, while ALDH1A3^-^ cells failed to create such structures. ALDH1A3^+^ cells also exhibit characteristics of CSCs and are resistant to p53-induced apoptosis 194. Xu et al. reported that the endothelial marker TEM8 was highly expressed in BCSCs and that TEM8^+^ breast cancer cells represented a kind of special BCSC to initiate VM 86. Tiara et al. developed a TEM8-specific CAR-T immunotherapeutic strategy to target TEM8^+^ endothelial cells and TNBC cells. They found that TEM8^+^ endothelial cells were killed and neovascularization was blocked and CD24^-^/CD44^+^ BCSC numbers were reduced, offering preclinical proof for immunotherapeutic targeting of tumor vascularization 195. However, we cannot determine whether there is a single subtype of BCSCs in tumors or whether BCSC subpopulations are involved because the marker of CSCs involved in VM formation varies in different studies.

Endothelial cells also support BCSCs independent of their vascular functions. Pegah et al. found that the NOTCH ligand Jagged1 was secreted by endothelial cells and activated the NOTCH pathway in BCSCs; thus, endothelial cells conferred a survival advantage and metastatic potential for BCSCs. Esak et al. cocultured MDA-MB-231 cells with lymphatic endothelial cells (LECs), microvascular endothelial cells (MECs), or human umbilical vein endothelial cells (HUVECs) and found that only LECs supported tumor cell growth. They discovered that tumor-educated LECs secreted amounts of epidermal growth factor (EGF) and PDGF-BB to promote tumor growth 196.

#### Immune cells

The interaction between the host immune system and cancer progression consists of three stages: clearance, balance, and escape. During the balance phase, CSCs might be the first batch of tumor cells that escape immune surveillance. Benefiting from a long quiescent stage, CSCs are slippery and rarely targeted by the immune system even when they enter the bloodstream and are surrounded by immune cells. In addition, BCSCs express low levels of MHC class I molecules and defects in antigen processing to escape immune cell killing 197, 198. BCSCs also secrete cytokines to suppress the activation of immune cells, including TGF-β, IL4, IL10 and IL33 199, 200. Jiang et al. employed single-cell analysis to uncover the dynamic change in immunity for heterogeneous BCSCs during tumor progression. They found that C-X-C motif chemokine ligand-16 (CXCL16) and CXCL1 were highly expressed in one of five BCSC clusters. Their corresponding receptors, CXCR2 and C-X-C motif chemokine receptor 6 (CXCR6), were also abundant in macrophages and T cells, respectively, suggesting communication between BCSCs and immune cells 201.

A large number of studies has reported the association between TAMs and poor clinical outcome. CSCs in breast cancer escape innate immune surveillance by upregulating the expression of CD47 202. Cancer stem cells promote macrophage M2 differentiation, which has protumoral and immune-suppressive functions, while the interaction between CSCs and macrophages elevates ALDH activity and chemoresistance 203, 204. Targeting colony stimulating factor 1 receptor (CSF1R) or C-C motif chemokine receptor 2 (CCR2) relieved immunosuppression, decreases the proportion of CSCs, and sensitizes tumors to chemotherapy and immunotherapy 205-207.

MDSCs are a heterogeneous population of immunosuppressive cells. The high number of MDSCs in circulation is related to tumor progression, including metastasis. By depleting required amino acids, MDSCs restrict T-cell proliferation and suppress T-cell function 208. Wei et al. observed a linear correlation between MDSC expansion and large tumor size 209. MDSCs also confer cancer cells with stem-like characteristics and EMT through IL6- and NO-mediated STAT3 and NOTCH pathway activation 210. Kumar et al. observed that TNBC cells recruited MDSCs via ΔNp63-dependent secretion of CXCL2 and CCL22. Meanwhile, recruited MDSCs secreted MMP-9 and chitinase 3-like 1 to promote TNBC stemness 211.

Although the infiltration of T cells is a favorable index of chemotherapy response and patient survival, several studies revealed that Tregs promote BCSCs. Roland et al. observed that CD8^+^ T cells upregulated the expression of stemness genes and immune checkpoint genes 212. Cells that preferentially exclude DNA binding dye Hoechst 33342 are more capable of initiating tumors and were called side population according to its unique pattern on fluorescence-activated cell sorting (FACS) analysis 213. It was reported that Foxp3^+^ Tregs increased the proportions of side populations and ALDH^+^ BCSCs in three mouse breast tumor cell lines 214. They also discovered that SOX2 promoted the expression of p65 and CCL1 in BCSCs to recruit Tregs 214. Indoleamine 2,3-dioxygenase (IDO) is an enzyme responsible for the degradation of the essential amino acid tryptophan and is highly expressed in BCSCs 215. IDO secreted by BCSCs suppresses cytotoxic T-cell expansion and promotes Treg activation 216. Nevertheless, different T-cell subsets recruited by BCSCs facilitate them maintenance of an immunosuppressive microenvironment.

## Strategies targeting BCSCs

### Natural compounds and their products

Nature is a treasure trove of natural compounds produced quantitatively through synthetic biology. It is a candidate drug for treating BCSCs (**Table 1**). Baicalein (5,6,7-trihydroxy-2-phenyl-4H-1-benzopyran-4-one) is an active ingredient of the roots of *Scutellaria baicalensis Georgi*. Baicalein reverses the drug resistance of MDA-MB-231/IR cells by upregulating interferon induced protein with tetratricopeptide repeats 2 (IFIT2) to induce the apoptosis of BCSCs 217. Phenethyl isothiocyanate (PEITC) can eliminate CSCs in MDA-MB-231/IR cells by reducing glutathione levels and promoting ROS accumulation and significantly inhibited the formation of mammospheres 218. Psoralidin, an active compound extracted from *Psoralea corylifolia*, targets NOTCH-1 in BCSCs and inhibits EMT and breast tumor growth 219.

### Antibody-based biopharmaceuticals

Antibodies have therapeutic effects on cancer by highly specifically targeting tumor cell surface antigens and immune cells, but their killing in tumor cells is limited 233. Because of its wide, “off-the-shelf” availability to become promising biopharmaceuticals 234. mAb4C5 is a monoclonal antibody that inhibits the colony formation of MDA-MB-231-derived BCSCs and reduces primary tumor growth by binding to extracellular heat shock protein 90 (HSP90) 235. The monoclonal antibody 602.101 specifically recognizes NOTCH-1, inhibits the expression of downstream target genes, reduces the MDA-MB-231 cancer stem-like cell subpopulation, inhibits the efficiency of mammosphere formation, and induces apoptosis 236. Monoclonal antibody J1-65D against NOTCH ligand jagged 1 (JAG1) reduces MDA-MB-231 BCSC numbers and breast tumor growth in a xenograft model by blocking NOTCH signaling 237. In MDA-MB-231 and T47D cells, the CD47 antibody B6H12 significantly downregulates KLF4 levels and negatively regulates EGFR phosphorylation to prevent BCSC proliferation, thereby inhibiting tumor growth 238. Dinutuximab, an anti-GD2 antibody, inhibits BCSC function and induces TNBC apoptosis via antibody-dependent cell-mediated cytotoxicity 239. The rhoptry protein 1 (ROP1)-specific antibody cirmtuzumab effectively attenuates chemotherapy-resistant BCSCs by reverting stemness 240. An anti-Cadherin 11 (CDH11) monoclonal antibody attenuates CSC-like properties and breast cell metastasis by upregulating miRNA-335 241. A single-domain antibody can block the interaction of PROCR with its ligands, effectively reducing BCSCs without tumor recurrence 242. John et al. synthesized antihuman CD133 scFv-PE38KDEL to kill CSCs and inhibit tumor growth in breast cancer 243. Not unique but has a double, CD133-targeted polymeric nanoparticles effectively impair the mammosphere formation ability of CSCs via conjugating anti-CD133 monoclonal antibody 244.

### Cytotherapy

The efficacy of cytotherapy in removing malignant tumor cells is encouraging and can prolong the survival of patients. Its biggest advantage is that enhances or alters intrinsic immune capacity, but there are fatal adverse reactions 245, 246. Third-generation cytotherapy is a combination of dendritic cells and cytokine-induced killer cells (DC-CIKs). Chen et al. reported that total RNA-loaded DC-CIK immunotherapy is extremely effective in counteracting BCSCs 247. TEM8 CAR-T can kill BCSCs, inhibit mammosphere formation, eliminate tumor angiogenesis, and induce PDX regression 195. GD2 has also been identified as a BCSC-related marker 248. GD2-CAR-T immunotherapy has site-specific activation, which can eliminate BCSCs and prevent the formation of metastasis 249.

### Synthetic small molecule compounds

Small molecule compounds have significant advantages such as the possibility of oral administration, stability, membrane permeability and non-immunogenicity in spite of off-target toxicity. Its convenient production has higher expected benefits 250-252. Currently, several synthetic compounds inhibit BCSCs (**Table 2**). Palbociclib is an oral biologically effective second-generation CDK4/6 inhibitor approved by the FDA that induces cell cycle arrest in breast cancer. Palbociclib inhibits MCF-7 BCSCs and effectively slows the formation of 3D spheroids 253. Protein arginine methyltransferase 5 (PRMT5) was reported to stabilize KLF4/5 proteins in breast cancer 254, 255. WX2-43 specifically blocked PRMT5-mediated KLF4 methylation and inhibited MDA-MB-231 BCSC activity 256. Consistently, the PRMT5 inhibitor PJ-68 efficiently promotes KLF5 degradation and inhibits stemness in BLBC 255. Ladademstat, an LSD1 inhibitor, blocks SOX2 activation, thus significantly reducing the number of mammospheres 257.

### New uses of old drugs

With the increasing difficulty of drug development, new uses of conventional drugs have broad application prospects. It is highly safe and may reduce overall development costs and shorten development time, but its use in cancer is still affected by multi-drug resistance mechanisms 268. The antilipemic agent lovastatin restores the sensitivity of triple-negative BCSCs to tyrosine kinase-targeted drugs by inducing HER2 expression and the stress response pathway 269. Salinomycin decreases NANOG, OCT4, SOX2, and glioma-associated homolog-1 (GLI-1) expression levels to attenuate the BCSC ratio 270, 271. Interestingly, the salinomycin derivative ionomycin also shows the ability to kill BCSCs 272. Disulfiram, a drug for alcoholism, inhibits breast cancer cell stemness by upregulating miR-30a to target SOX4, thereby blocking the TGF-β/SMAD pathway 273. As a first-line drug for diabetes, metformin partially reduces the percentage of TNBC stem cells through the PKA-GSK3β-KLF5 signaling pathway 152. In addition, metformin kills MCF-7 stem cells, and its medicinal properties are improved by enhancing AMPK activation in combination with hyperthermia 274. Mifepristone, an abortifacient, inhibits KLF5 expression by inducing the expression of miR-153, thereby reducing the TNBC stem cell population 275. Furthermore, the mifepristone derivatives FZU-00,003 and FZU-00,004 exert anticancer activities 276, 277. Flubendazole is a clinically anthelmintic that exhibits potential anti-tumor activity by reducing the expression of CD49f and ALDH1* in vitro* and* in vivo* and killing BCSCs cells 278.

### Nucleic acid medicines

Nucleic acid medicines have achieved a short reaction time, long-lasting therapeutic effect, minor side effects, and high specificity. Still, their shortcomings are lacking available genetic information and limited transmission to target organs and cells. Once these urgent problems are solved, chemical synthesis can significantly improve its application ability 279, 280. Cripto-1 is the downstream target of NANOG and OCT4. The Cripto-1 encoding DNA vaccine can target BCSCs, reduce breast cancer metastasis and improve the survival rate 281. Cystine-glutamic acid reverse transport protein xCT (SLC7A11) is overexpressed in BCSCs. The antibody produced by the anti-xCT virus vaccine directly impaired BCSCs and weakened tumor growth and metastasis 282. The complex composed of an RNA aptamer targeting epithelial cell adhesion molecules and survivin siRNA was delivered to BCSCs to induce apoptosis, inhibiting tumor growth 283.

Currently, there are several strategies to target BCSCs, each with its own advantages and disadvantages **(Table 3)**. So far, there are a variety of drugs have been introduced into the clinical to bring great cure hope to patients. However, the problem is that breast cancer will develop resistance to certain drugs, and one of the reasons is the presence of CSCs. There are also a variety of therapies targeting CSCs. CAR-T therapy in immunotherapy has attracted much attention due to its high efficiency in killing cancer cells, but its fatal disadvantage is that it cannot distinguish the expression level of the same target protein in different cells, which will lead to strong side effects. At present, there are several difficulties in the treatment of CSCs. Firstly, it is difficult to distinguish the molecular characteristics of normal stem cells and CSCs. They share cell surface markers and signaling pathways, and the specificity of CSCs remains to be explored. Secondly, it is necessary to use a variety of combined treatments to extinguish the dormant and proliferating CSCs. Finally, if the CSCs can be eliminated early in the tumor, then the treatment effect will be more effective.

## Conclusions and perspectives

Current breast cancer therapies have significantly improved in eliminating primary tumors and prolonging survival. However, effectively eliminating BCSCs is difficult. First, the dynamic intrinsic property of BCSCs and the unique CSC niche make it challenging to eradicate BCSCs. Second, BCSCs can switch their state between dormancy and rapid reproduction. Killing cells in a quiescent state is still a challenge. Third, the unique microenvironment of BCSCs is not well elucidated, and most current *in vivo* studies on BCSCs are performed in an immune-deficient environment, making it challenging to recapitulate biological complications in the clinic.

Although the single-cell sequencing studies did not detect a single distinct stem cell population with unique transcriptional features in the adult murine gland, these sequencing data provided a detailed transcriptional map of mammary epithelial differentiation, suggesting that scRNA-sequencing coupled with flow cytometry might be a powerful tool for CSC discovery and isolation. In addition, targeting unique regulatory factors that contribute to maintaining CSC niches in combination with traditional treatment against rapidly proliferating cancer cells will be a promising approach and has shown synergistic effects. However, the safety and efficacy of the combined strategies need to be evaluated in preclinical and clinical studies. Modulating the tumor microenvironment to target BCSCs also exhibited encouraging results. At present, there are various therapies for BCSCs, each with its own advantages and disadvantages. In addition to the drugs mentioned above, EpCAM-CAR-T (NCT02915445) has entered the clinical state for BCSCs immunotherapy, and good results are expected. Currently, only cervical cancer vaccine has entered clinical application, specific antigens for BCSCs to develop antibodies against breast cancer need further investigation. With increasing progress in BCSC research, patients will benefit from BCSC-based personal cancer treatment.

## Funding

This work was supported by National Key R&D Program of China (2020YFA0112300 and 2020YFA0803200), National Natural Science Foundation of China (U2102203 and 81830087), and Yunnan Fundamental Research Projects (202101AS070050). “Ten Thousand Plan” - National High-Level Talents Special Support Plan (WR-YK5202101); Program for Outstanding Leading Talents in Shanghai; Program of Shanghai Academic/Technology Research Leader (20XD1400700); Program for Outstanding Medical Academic Leader in Shanghai (2019LJ04); The innovative research team of high-level local university in Shanghai.

## Figures and Tables

**Table 1:** Natural pharmaceutical products targeting BCSCs

Names	Target	Effects of a model	Ref.
Triterpene acid	C-MYC	Inhibits MDA-MB-231 stemness and the formation of mammospheres.	220
Gomisin M2	Wnt/β-catenin	Decreases the proliferation of TNBCs and mammosphere formation.	221
Honokiol	STAT3	Reduces of secondary transplanted tumors and the prolongation of metastasis time of MDA-MB-231 xenografts.	222
Apigenin	YAP/TAZ-TEAD	Inhibits TNBC stemness, migration, and tumor growth.	223
Andrographolide	Survivin	Inhibits the activity of human BCSCs in a dose-dependent manner and reduces the formation of mammospheres.	224
Ellagic acid	Actinin alpha 4 and β-catenin	Inhibits MDA-MB-231 and BT549 cell proliferation and prolongs the survival time of MMTV-PyMT mice.	225
8-Hydroxydaidzein	JAK2 and STAT3	Decreases BCSC characteristics and triggers apoptosis.	226
Sulforaphane	Wnt/β-catenin	Eliminates BCSCs and abrogates tumor growth.	227
Withaferin A	BMI-1	Attenuates BCSCs and inhibits breast tumor burden.	228
Benzyl isothiocyanate	Ron receptor tyrosine kinase	Inhibits the self-renewal ability of BCSCs.	229
Glabriden	TGF-β/SMAD2	Attenuates the breast CSC-like properties.	230
Isoliquiritigenin	DNA methyltransferase 1 (DNMT1)	Reduces the BCSC-like population and suppresses breast cancer initiation and progression.	231
Caffeic Acid Phenethyl Ester	CD44	Interfering with the growth of TNBC CSCs.	232

**Table 2:** Synthetic compounds targeting BCSCs

Name	Targets	Effect of the model	Ref.
WX2-43	Blocks PRMT5-mediated KLF4 methylation	Inhibits MDA-MB-231 BCSC activity.	256
PJ-68	Promotes KLF5 degradation	Inhibits stemness in BLBC.	255
B591	mTOR	Reduces the self-renewal ability of MCF-7, SUM159PT and MBA-MB-231 and tumorigenesis.	258
Pyrvinium pamoate	Cholesterol and fatty acid synthesis	Inhibits SUM159PT tumor growth and metastasis.	259
AZD1775	Inhibits mucin 1 (MUC1) expression and cell cycle arrest	Reduces the BT474 CSC percentage and inhibits tumor growth.	260
Tannic acid	p65-IL6	Decreases the formation of mammospheres in MCF-7, T47D and MDA-MB-231 cells.	261
Propofol	PD-L1 and NANOG	Decreases MCF-7 and MDA-MB-231 CSC mammosphere forming ability.	262
Dodecyl-TPP	mitochondria	Reduces cell viability and mammosphere formation.	263
Ferutinin analog	ERα	Induces apoptosis and the anti-proliferative activity of BCSCs.	264
Lx2-32c	FoxM1 and CD44	Decreases MDA-MB-231 derived cancer stem cell-like characteristics.	265
108600	CK2/DYRK/TNIK	Has a curative effect on BCSCs.	266
Doxycycline	CD44 and ALDH1	Likely extinguishes BCSCs.	267

**Table 3:** Advantages and disadvantages of therapeutic strategies

Strategy	Advantages	Disadvantages	Ref.
Natural compounds	Have biological activity and high bioavailability.	Separation, synthesis and the target protein analysis are difficult.	284
Antibody-based biopharmaceuticals	Have highly specific targeting tumor cell surface antigens and immune cells.	Its killing in tumor cells is limited.	233, 234
Cytotherapy	Enhance or alter intrinsic immune capacity.	There are fatal adverse reactions.	245, 246
Synthetic small molecule compounds	Have the possibility of oral administration, stability, membrane permeability.	Has off-target toxicity.	250-252
New uses of old drugs	Highly safe and may reduce overall development costs and shorten development time.	Is still affected by multi-drug resistance.	268
Nucleic acid medicines	Achieved a short reaction time, minor side effects, high specificity and long-lasting therapeutic effect.	Are lacking available genetic information and limited transmission.	279, 280
